# Preparation of N,S-codoped magnetic bagasse biochar and adsorption characteristics for tetracycline

**DOI:** 10.1039/d1ra08404f

**Published:** 2022-04-19

**Authors:** Wu Junfeng, Hou Bowen, Wang Xiaoqing, Liu Zuwen, Wang Zhaodong, Liu Biao, Li Songya, Gao Hongbin, Zhu Xinfeng, Mao Yanli

**Affiliations:** Henan Key Laboratory of Water Pollution Control and Rehabilitation Technology, Henan University of Urban Construction Pingdingshan 467036 China; School of Architectural and Surveying & Mapping Engineering, Jiangxi University of Science and Technology Ganzhou 341000 China 469346791@qq.com; Henan Province Town of Comprehensive Design and Research Institute Pingdingshan 467036 China

## Abstract

Agricultural waste disposal and purification of polluted water are always the key issues of environmental restoration. In this work, thiourea-functionalized magnetic bagasse biochar (MFeBC) was prepared for tetracycline (TC) removal from aqueous solutions. Firstly, MFeBC was prepared by a combined impregnation and chemical coprecipitation method. Furthermore, MFeBC was characterized by Brunauer–Emmett–Teller surface area analysis, Fourier transform infrared spectrometry, X-ray diffraction analysis, scanning electron microscopy, X-ray photoelectron spectroscopy and the magnetic hysteresis curves. For the TC adsorption, the effects of different solution pH level, adsorbent dosage, initial TC concentration and temperature on the adsorption performance were studied respectively. Moreover, the results indicated that the Freundlich isotherm models appropriately described the adsorption process. The kinetic data were better fitted by the pseudo-second-order kinetic model. The maximum TC adsorption capacity of MFeBC reached 69.26 mg g^−1^. Hydrogen bonding and Π–Π interactions played a dominant role in the adsorption process. Therefore, MFeBC can be used as an effective adsorbent for tetracycline removal from aqueous solution.

## Abstract 2

Preparation of N,S-codoped magnetic bagasse biochar and adsorption of tetracycline.

## Graphical abstract

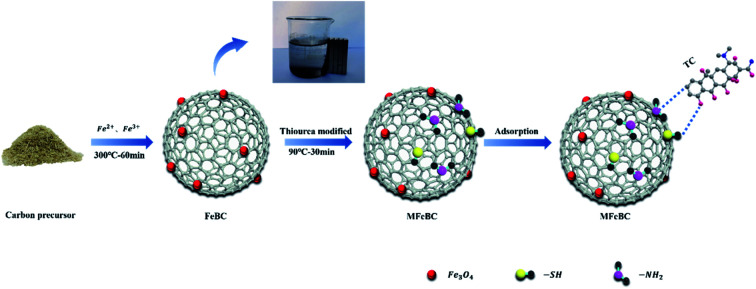

## 1. Introduction

With the increasing consumption of antibiotics year by year, antibiotics pollution problems and the potential hazards have attracted many domestic or foreign researchers.^1^ Among them, TC antibiotics are a broad-spectrum class of antibiotics discovered in the 1940s and widely used against infections caused by Gram-positive and Gram-negative bacteria, intracellular mycoplasma and chlamydia.^2^ Due to its stable structure containing naphthalene, TC can hardly be metabolized by animals.^3^ 70–90% of TC antibiotics is excreted to the environment, posing potential threats (acute or chronic) to human health.^4^ Therefore, tetracycline (TC) has one of the highest concentrations and frequency detection in aquatic environments.^5^ Thus, an effective handing of TC is of great significance.

Many efforts have been made to effectively remove TC from wastewater, such as advanced oxidation,^6^ photocatalytic degradation,^7^ electrochemical treatment,^8^ membrane separation,^9^ biodegradation^10^ and adsorption.^11,12^ Among these methods, adsorption is considered to be an economic and effective method for its' advantages of convenient operation, high removal efficiency, no secondary pollution and easy recycling.^13^ For traditional adsorbent materials were expensive, such as activated carbon and ion exchange resin, the research of industrial by-products or agricultural wastes as low-cost carbon-based adsorbent materials has been paid more and more attention. With fascinating characteristics, high electrical conductivity, environmental benignity, stable electrochemistry and large specific surface area, carbon-based materials have been widely used in the field of adsorption and supercapacitor.^14^

Sugarcane is widely used in sugar industry and a large amount of bagasse waste was produced.^15^ Bagasse mainly consists of cellulose (50%), polyoses (27%) and lignin (23%), so it is an excellent precursor for biochar preparation.^16^ Many studies have shown that bagasse can be used to remove different antibiotics.^17–19^ However, its adsorption capacities for antibiotics were poor, and more importantly it was very difficult to separate the adsorbent from solution in practical application. Therefore, how to improve its adsorption capacity, effectively recycle and utilize biochar is the current research hotspot.^20^

In recent years, magnetic separation technique has been developed and attracted much attention for its unique separation performance.^21^ Modified biochar with magnetic media (Fe, Co, Ni, or their oxides) could achieve rapid separation and recovery in an external magnetic field.^22^ There were two main methods of magnetic attachment, one was co-pyrolysis after impregnation with magnetic precursors,^23^ the other was chemical co-precipitation of ferric and ferrous ions on the surfaces of biochar under alkaline conditions.^24^ The magnetic intensity of biochar always affects the adsorption performance, so the innovative methods and the specific adsorption mechanism still needs to be further studied.

In addition to strong magnetic, the strength of adsorption performance was the fundamental factor to measure the performance of biochar. Thiourea is a kind of non-pollution and low-toxicity reagent rich in nitrogen and sulfur.^25^ Many studies have shown that thiourea modification can improve the adsorption properties of biochar for many pollutants.^26–28^ Wei Mao *et al.*,^29^ used thiourea to introduce N–S elements to the surface of corn stalk biochar, the adsorption performance of ciprofloxacin and Cr was improved greatly. Therefore, thiourea can be a potential modifier for preparation of high-performance magnetic biochar.

In this study, thiourea-functionalized magnetic bagasse biochar (MFeBC) was successfully prepared by combined co-pyrolysis and co-precipitation. The co-precipitation reaction was carried out on the carbon precursors, and the bagasse was impregnated with Fe^2+^/Fe^3+^ mixed solution, followed by calcination carbonization, which can further enhance the magnetism.^30^ And the effects of magnetic and thiourea-magnetic modifications on characteristics of biochar were explored. Then, the TC adsorption behavior, including the adsorption kinetics, isotherms and adsorption mechanisms were studied. This research provided important theoretical and technical support for the potential application of highly adsorption magnetic biochar for antibiotics.

## 2. Experiment part

### 2.1 Chemicals and materials

Bagasse was collected from a fruit market in Pingdingshan of Henan province, China. All chemicals including KOH, HCl, ethyl alcohol, thiourea, FeCl_3_ and FeSO_4_·7H_2_O were analytically pure reagents purchased from Sinopharm Chemical Reagent Co., Ltd (Shanghai, China). Tetracycline hydrochloride (TC) (purity ≥ 95%) was purchased from Sigma. The pH of the TC solution was adjusted by HCl or NaOH aqueous solution with suitable concentration.

### 2.2 Preparation of MFeBC

The collected bagasse was washed by distilled water and soaked for 24 h to remove excess sugar from the bagasse. Then dried in an oven at 80 °C for 24 h. Finally, they were crushed into small pieces and sifted.

Magnetic composite was synthesized in an ultrasound clean bath operating at 25 kHz with a power of 140 W (KQ-200KDE, China). 5 g screened bagasse was added to 200 mL solution (FeCl_3_: 0.05 mol L^−1^, FeSO_4_: 0.025 mol L^−1^). Then, ammonia water (3.5 mol L^−1^) was added dropwise into mixed Fe^2+^/Fe^3+^ solution until pH > 10.0. The magnetic bagasse was collected after 30 min reaction. The collected solid was rinsed repeatedly with distilled water to remove excess iron ions and makes the solution neutral. Then, the magnetic bagasse was calcined at 300 °C for 60 min to obtain magnetized bagasse biochar (FeBC). Particle between 100-mesh (0.15 mm) and 120-mesh (0.12 mm) was used.

At last, magnetic bagasse biochar was modified by thiourea. FeBC (1 g) was immersed in a mixed water solution (25 mL) with 0.0625 mol L^−1^ KOH, 0.0821 mol L^−1^ thiourea and 33% ethanol. Then the resultant suspension was heated in a water-bath at 90 °C for 30 min. After washing the product to pH = 7.0 and drying it in an oven, the final product magnetized modified biochar (MFeBC) was obtained.

### 2.3 Characterization of biochar

The specific surface area (*S*_BET_) was evaluated from the N_2_ adsorption isotherms with a surface area and porosity analyzer (Micromeritics ASAP 2020, America). The surface morphology was characterized using scanning electron microscopy (SEM, S-4800, Hitachi). The X-ray diffractometer (XRD, F-7000, Shimadzu) was used to detect the crystal structure of the biochar. Additionally, the functional groups on biochar were analyzed by Fourier Transform Infrared Spectroscopy (FTIR, NEXUS 670, Thermo Fisher Nicolet, USA). The surface chemical composition was confirmed by the X-ray photoelectron spectroscopy (XPS, Escalab 250xi, Thermo Fisher, USA). The magnetic properties of MFeBC were assessed using a vibrating sample magnetometer (SQUID-VSM, Quantum Design, USA).

### 2.4 Adsorption experiments

Batch adsorption experiments were carried out to study the adsorption behaviors of TC on MFeBC. The impact of different factors was studied, such as solution pH, adsorbent doses, contact time, initial TC concentration and temperature. In a typical run, adsorbent was added to the TC solution with different pH or TC concentration. The samples were taken out at different reaction time, and then the adsorbent was quickly separated from the solution by centrifuge. The residual TC concentration was detected by UV-vis spectrophotometer (TU-1810, Persee) at 271 nm. Each experiment was repeated three times to get the average.

The TC removal efficiency and TC equilibrium adsorption capacity were calculated according to the following equation eqn (1) and eqn (2):

1 *R* = (*C*_0_ − *C*_e_)/*C*_e_ × 100

2 *q*_e_ = (*C*_0_ − *C*_e_)*V*/*m*

where *R* (%) was the removal rate; *C*_0_ and *C*_e_ (mg L^−1^) indicated the TC initial concentration and TC equilibrium concentration; *q*_e_ (mg g^−1^) was the TC equilibrium adsorption capacity; *V* (L) was the initial TC volume; *m* (mg) was the mass of adsorbent.

## 3. Result and discussion

### 3.1 Biochar characterization

#### 3.1.1 XRD

**Fig. 1:**
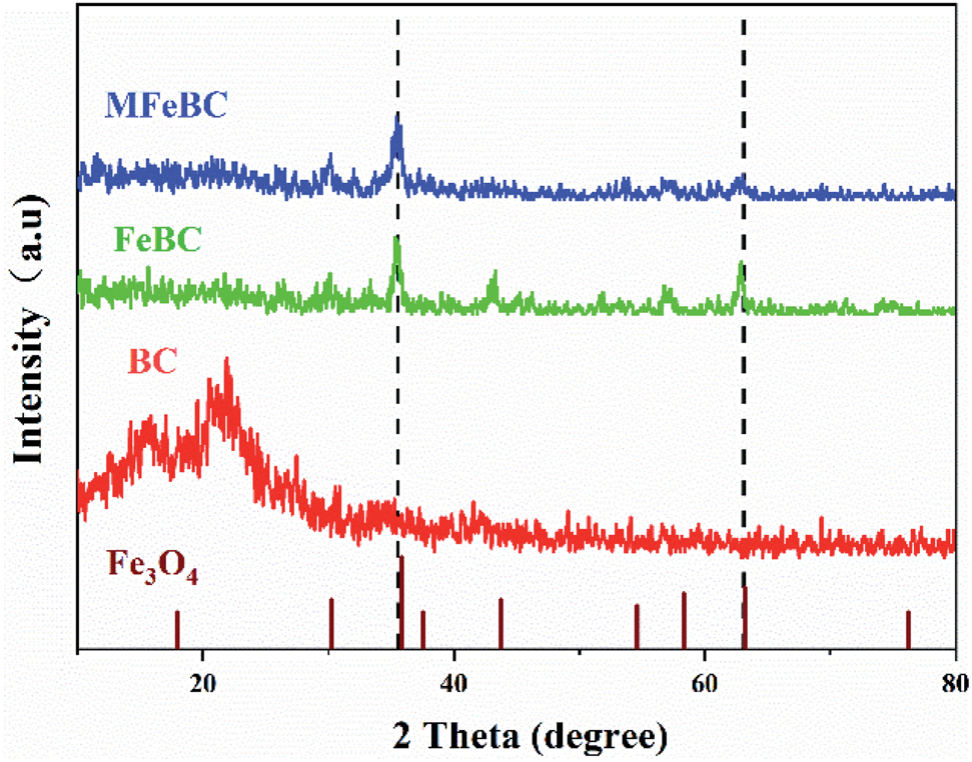
The XRD patterns of BC, FeBC and MFeBC.

The XRD patterns of BC, FeBC and MFeBC were presented in Fig. 1 to analyze the crystalline mineral structure of adsorbents. The broad peak around at 26.4° was graphitic carbon, which described their good carbonization degree during the pyrolysis process.^31^ Compared to BC, five new diffraction peaks around at 30.3°, 35.8°, 43.8°, 58.3°, and 63.2° were observed, which were indexed to (220), (311), (400), (511) and (400) planes of Fe_3_O_4_,^32,33^ based on the analysis of the software Jade 6.0 (JCPDS no. 26-1136). The XRD patterns of MFeBC and FeBC were identical and identified as Fe_3_O_4_. But the peak intensity reduced slightly, suggesting that thiourea modification will affect the crystal structure of Fe_3_O_4_ to a certain extent. In general, Fe_3_O_4_ was successfully loaded on the surface of biochar.

#### 3.1.2 SEM

**Fig. 2:**
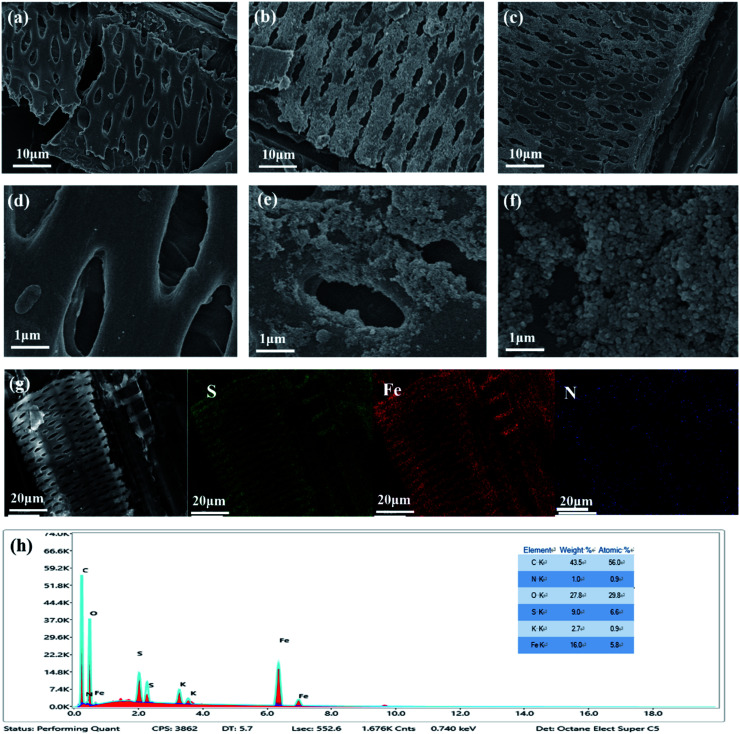
SEM images of (a and d) BC, (b and e) FeBC, (c and f) MFeBC, (g) mapping and (h) element ratio of MFeBC.

The surface morphology and pore structure have an obvious influence on adsorption performance. Further, it was very helpful for the research on the adsorption performance. In this research, the surface morphology and pore structure of BC, FeBC and MFeBC was analyzed, and the results were presented in Fig. 2. Using bagasse as the precursor material, its morphological structure with open macroporosity (Fig. 2(a)) allowed for the penetration of iron ions (both Fe^2+^ and Fe^3+^) into the inner pore space, as well as for deposition of these ions on the outside surfaces.^34^ The incorporation of iron ions occurred onto electron-rich oxygen atoms of the polar hydroxyl/ether groups in cellulose. This immobilized the iron ions onto the BG surfaces, both in the inner and the outer macropores.^19^ From Fig. 2(d), the surface of BC was relatively smooth and had no obvious particle coverage. After magnetization (Fig. 2(e)), a large number of Fe_3_O_4_ spherical particles were observed on the surface. In additional, more pores were formed (as Fig. 2(f)), which can be attributed to the load of Fe_3_O_4_ nano-particles. The conclusion of Zhou *et al.* and Jaegwan Shin *et al.*^35,36^ have also confirmed our point of view. For MFeBC, spherical particles can also be seen (Fig. 2(c)). Further, more pore structures developed pore in the surface of MFeBC. The presence of S and N in Fig. 2(h) was account for the modification of thiourea introducing amidogen and sulfur-containing groups to MFeBC. Therefore, it was confirmed that MFeBC contained C, N, O, S and Fe, which was matched with SEM mapping.

#### 3.1.3 BET

**Fig. 3:**
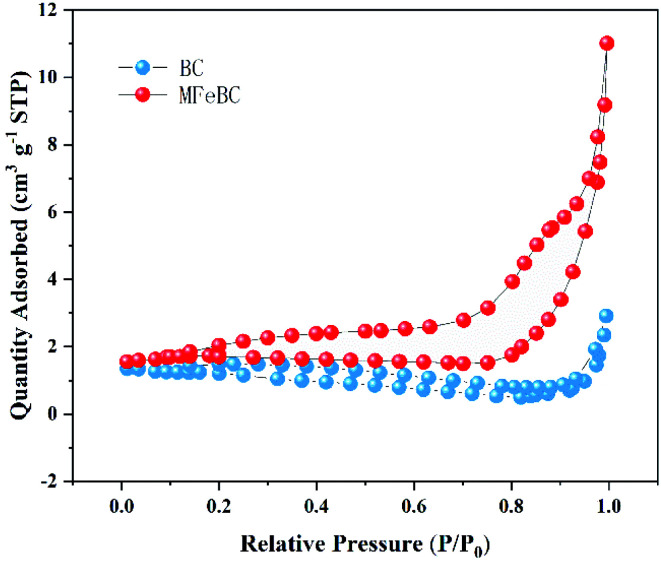
N_2_ adsorption/desorption isotherms of BC and MFeBC.

N_2_ adsorption–desorption method was carried out to study the porous structure of the original BC and MFeBC. As shown in Fig. 3, both curves displayed stable or slow downward trend at low relative press then a steep rise when *P*/*P*_0_ > 0.8. The adsorption of N_2_ decreases with the increase of *P*/*P*_0_, which may be due to the fact that the overall pore structure of the biochar calcined at low temperature (300 °C) was not developed and the material itself is fine powder particles (100–120 mesh). According to IUPAC classification,^37^ the isotherms of BC and MFeBC were exhibited as type II isotherms, indicating a large fraction of macropores. The specific surface area of BC and MFeBC reached 2.0710 m^2^ g^−1^ and 10.0525 m^2^ g^−1^, respectively. The Fe_3_O_4_ particles loaded on the surface of biochar can produce more pore structure, which was beneficial to the increase of specific surface area. Although the surface area of MFeBC was improved, the surface area was still at a low level. It was proved indirectly that the high adsorption of TC by MFeBC was due to the chemical adsorption rather than physical adsorption.

#### 3.1.4 FTIR

**Fig. 4:**
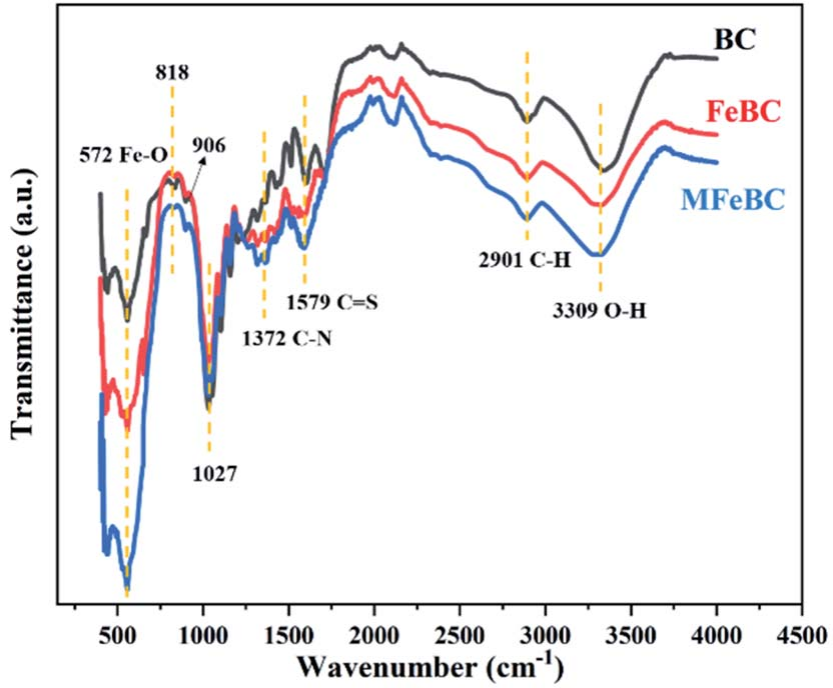
FTIR spectra of biochar before and after modification.

The functional groups in BC, FeBC and MFeBC were investigated by FTIR analysis (Fig. 4). After magnetization, a distinctive IR peak of the Fe–O band in relation to Fe_3_O_4_ was detected at 572.4 cm^−1^ in FeBC and MFeBC.^38^ The adsorption peaks at around 818.1 cm^−1^ and 906.0 cm^−1^ were due to the presence of aromatic/heteroaromatic structures,^39^ which could provide π-electron and had the potential to capture TC ions.^40^ The peak at 3309 cm^−1^ corresponded to the O–H stretching vibration of –OH.^41^ For MFeBC, the peak was wider and stronger, which due to the existence of hydrogen bond between –OH and –NH_2_.^42^ The peaks at 1372 cm^−1^ and 1579 cm^−1^ were associated with C–N bonding and C=S of thiourea moiety, respectively.^25^ These observations confirmed that MFeBC was successfully modified with thiourea. The other peaks were found at 2901 cm^−1^ (CH-stretching of aliphatic groups)^43^ and 1312 cm^−1^ (C–H bending of aldehyde groups). From the above results, abundant oxygen polar functional groups produced in MFeBC and they can facilitate the π–π conjugation between aromatic rings of biochar and TC.^44^

#### 3.1.5 XPS analysis

**Fig. 5:**
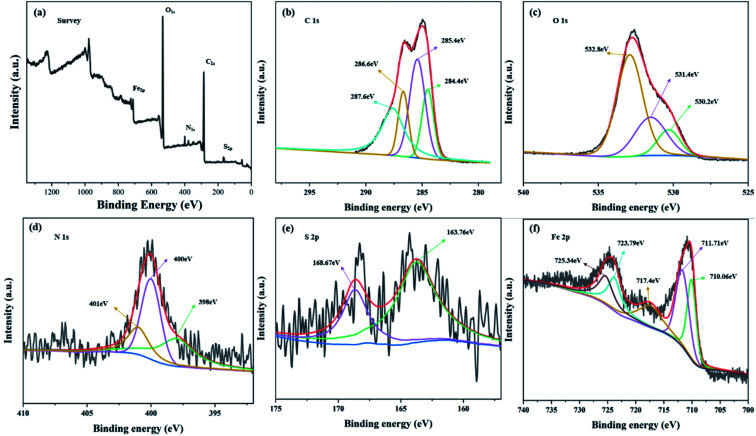
XPS spectra of MFeBC (a) survey (b) C 1s (c) O 1s (d) N 1s (e) S 2p and (f) Fe 2p.

High resolution XPS spectra of C, O, N, S, Fe and survey spectra of MFeBC are exhibited in Fig. 5. A dominant C 1s peak (∼285.0 eV), an O 1s peak (∼533.0 eV), a N 1s peak (∼400.0 eV), a S 2p peaks (∼165.0 eV) and a Fe 2p (∼710.0 eV) can be observed in the survey spectrum (Fig. 5(a)), confirming the existence of N, S and Fe. The high-resolution spectrum of C 1s shown that C existed in many forms in MFeBC (Fig. 5(b)). The strongest peak located at 285.4 eV and referred to C–O (alcoholic, phenolic, hydroxyl and/or other). The peak at 284.4 eV represented for C=C^45^ or C–C bonds.^46^ The peaks for C=O^47^ and C–N^48^ were found at 287.6 eV and 286.6 eV respectively. The deconvoluted results of the high-resolution N 1s spectrum shown in Fig. 5(d) revealed that there were three types of N species in MFeBC, corresponding to graphitic-N (401.0 eV), pyrrolic-N (400 eV), and pyridinic-N (398 eV).^49^ It has been reported that N can significantly increase adsorption capacity and diffusivity of biochar by facilitating the capacity and rate performance of material anode.^50^ In S 2p spectrum (Fig. 5(e)), 163.76 eV and 168.67 eV refer to C–S and RSO_3_^−^.^51^ Therefore, two types of sulfur containing groups were added on the surface of MBC: one was C–S formed through dehydration condensation of –SH in thiourea and –COOH on the surface of biochar, the other were small amounts of RSO^3−^ formed during this process. Meanwhile, five peaks were found in Fe 2p spectrum (Fig. 5(f)) including Fe^2+^ 2p_3/2_ peak at 710.06 eV, Fe^3+^ 2p_3/2_ peak at 711.716 eV, Fe^2+^ 2p_1/2_ peak at 723.79 eV, Fe^3+^ 2p_1/2_ peak at 725.34 eV, and a satellite peak at 717.4 eV were found and the results indicated that the Fe element in the MFeBC existed in the form of Fe^2+^ and Fe^3+^ simultaneously.^52^ The elemental analysis showed that the magnetic and functional modification of BC were successfully modified by Fe_3_O_4_ and thiourea.

#### 3.1.6 Magnetic properties

**Fig. 6:**
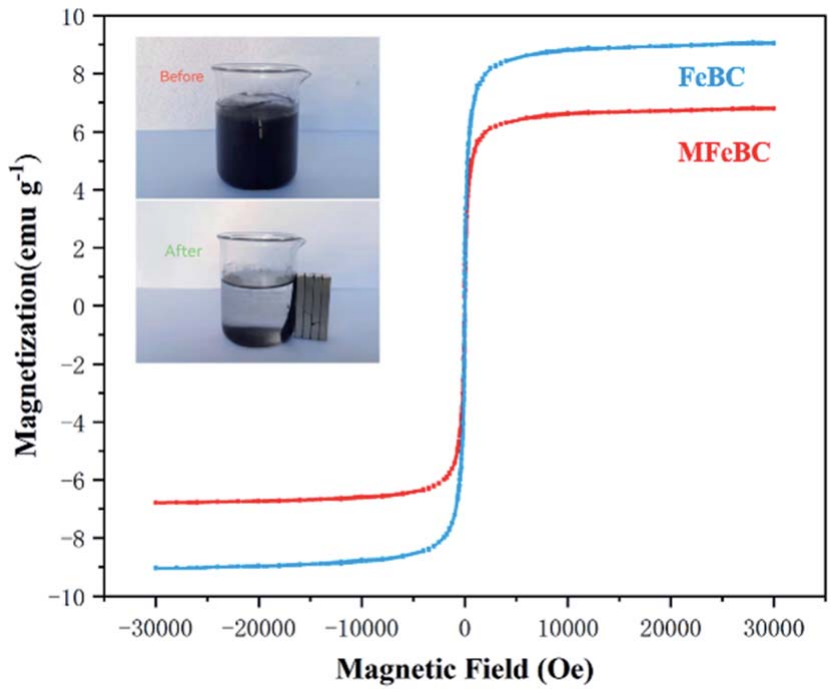
Magnetic hysteresis curves of MFeBC.

The magnetic behaviors of FeBC and MFeBC were studied using the vibrating sample magnetometer (VSM) at 25 °C. Fig. 6 presented the magnetic hysteresis curves of FeBC and MFeBC under an external magnetic field ranging from −30 000 to +30 000 Oe. The saturation magnetization values of FeBC and MFeBC were 9.12 and 6.86 emu g^−1^, respectively. The leaching of Fe during thiourea modification led to a slight decrease in saturation magnetization. Both materials have good superparamagnetism without coercive force and remanence.^53^ Besides, biochar well dispersed in water can be rapidly attracted by ordinary magnets and make the suspensions clear (inset in Fig. 6), which indicated that Fe_3_O_4_ has formed a good combination with biochar. This was consistent with the surface topography observed by SEM. These results further confirmed the excellent magnetic properties of the prepared magnetic biochar samples and could be fast separated and reused.

### 3.2 Different impacts

#### 3.2.1 pH

**Fig. 7:**
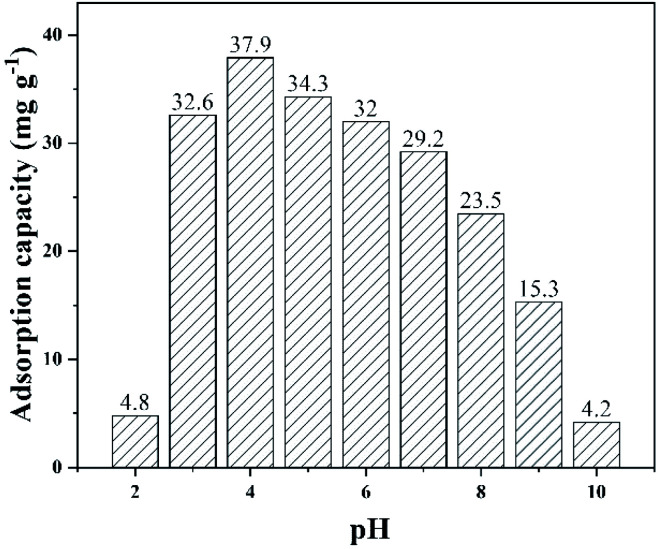
Effect of pH on adsorption capacity of TC using MFeBC (MFeBC = 0.5 g L^−1^, initial TC concentration = 50 mg L^−1^, contact time = 2 h, temperature = 25 ± 1 °C).

Solution pH can affect ionization degrees, surface charges of biochar, and the speciation of TC.^54^ The different initial pH (from 2.0 to 10.0) was adjusted using HCl (0.1 mol L^−1^) or NaOH (0.1 mol L^−1^) and the results were shown in Fig. 7. As the pH increased from 2.0 to 4.0, TC adsorption capacity increased. During 4.0 and 7.0, TC adsorption capacity was basically stable. However, TC capacity adsorption decreased rapidly as the pH further increased from 7.0 to 10.0. TC has three p*K*_a_ values (3.3, 7.7 and 9.7). Under pH < 3.3, 3.3 < pH < 7.7, 7.7 < pH < 9.7 and pH > 9.7, the species were H_4_TC^+^, H_3_TC, H_2_TC^−^ and HTC^2−^, respectively.^55^ Above 7.7, anion form was dominant and biochar surface was typically negatively charged.^56^ Electrostatic repulsion led the adsorption capacity reduction. During the pH range of 4.0 to 7.0, neutral ion form (H_3_TC) was dominant and electrostatic interaction may not be the cause of the high TC adsorption. Suggesting that other adsorption mechanisms may be present, such as H-bonding,^57^ Π–Π interaction^58^ and pore-filling.^59^ Overall, MFeBC had a higher TC adsorption effect in the pH range of 3.0–7.0.

#### 3.2.2 Adsorbent dosage

**Fig. 8:**
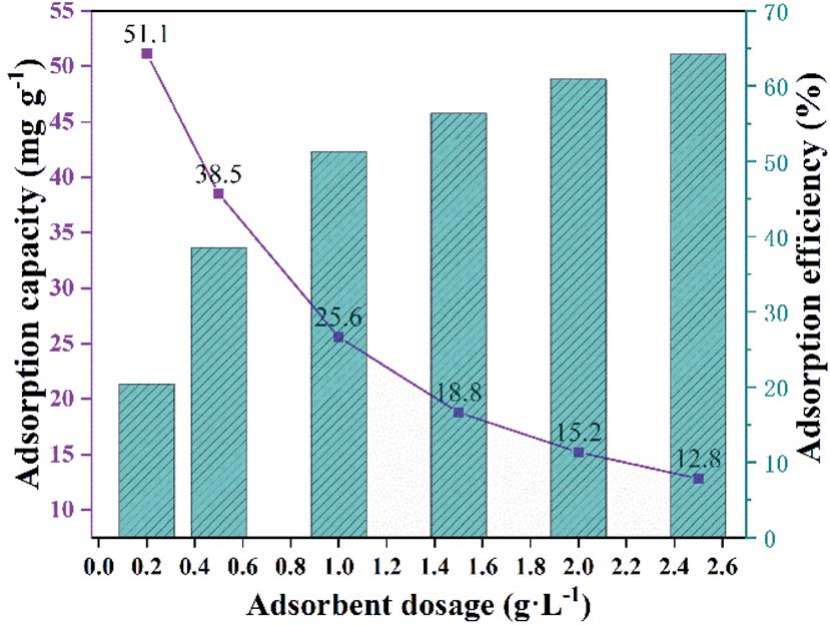
Effect of initial adsorbent dosage on adsorption capacity and adsorption efficiency of TC using MFeBC (initial TC concentration = 50 mg L^−1^, pH = 5.0, contact time = 2 h, adsorption temperature = 25 ± 1 °C).

Adsorbent dosage was a significant influence factor. The influence of MFeBC doses (0.2–2.5 g L^−1^) was studied at 25 °C and TC concentration of 50 mg L^−1^. As shown in Fig. 8, the TC removal efficiency increased from 20.4% to 64.2% with the adsorbent dosage increased from 0.2 to 2.5 g L^−1^. It was suggested that more MFeBC provided more accessible adsorption sites. In addition, the maximum adsorption capacity of 51.1 mg g^−1^ was obtained at the dosage of 0.2 g L^−1^.

### 3.3 Adsorption kinetics

**Fig. 9:**
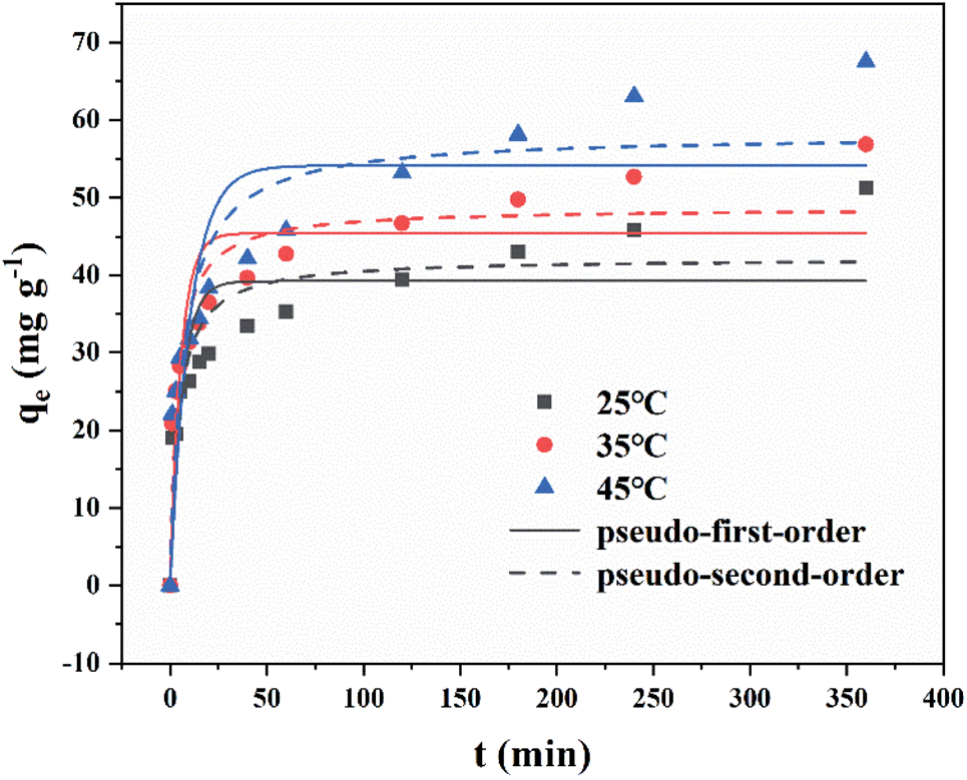
The adsorption kinetic plots of MFeBC in 25 °C, 35 °C, 45 °C (initial TC concentration = 50 mg L^−1^, pH = 5.0).

Adsorption rate and adsorption capacity were the key parameters to evaluate the performance of adsorbents. Therefore, studying the adsorption kinetics was very necessary since they indicated reaction rate, providing the information on the factors affecting the reaction rate and revealed adsorption mechanisms.^60^ Adsorption kinetic was researched at different temperature (25 °C, 35 °C, 45 °C), under the condition of 0.025 g of MFeBC and 50 mL of TC (50 mg L^−1^). Fig. 9 showed the adsorption kinetics of TC in different temperature. Obviously, the adsorption of TC present two stages. The first stage (0–30 min) was a rapid adsorption process due to the initial high TC concentration and multiple adsorption sites in biochar.^61^ In the second stage (above 30 min), the adsorption capacity increased in a slower rate and finally reached an equilibrium state. At different temperatures, the adsorption kinetics curve has the same trend. From Fig. 9, the maximum adsorption capacity increased as the temperature increased. This indicated that the higher temperature was conducive to the adsorption reaction.

To predict the TC adsorption mechanism by MFeBC, the adsorption process was tested with the pseudo-first-order (eqn (3)) and pseudo-second-order models (eqn (4)), respectively. Related parameters were listed in Table 1.

3 *q*_*t*_ = *q*_e_(1 − e^−*k*_1_*t*^)

4
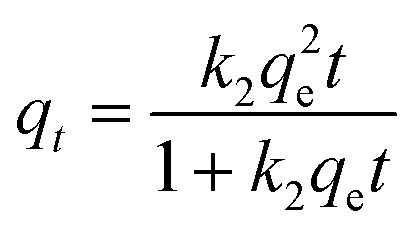
where *q*_e_ and *q*_*t*_ (mg g^−1^) correspond to the adsorption capacity at equilibrium and time *t*, respectively. *k*_1_ (min^−1^) and *k*_2_ (g mg^−1^ min^−1^) were the rate constants of the pseudo-first-order and pseudo-second-order adsorption, respectively. As shown in Fig. 9, the pseudo-second-order kinetic model was more consistent with the actual adsorption data. The regression coefficients *R*^2^ of the pseudo-second-order models (0.845, 0.885, 0.854) were higher than that of the pseudo-first-order models (0.702, 0.752, 0.720). Thus, it can be concluded that chemisorption was dominant in the adsorption process.

**Table 1:** Kinetics parameters of pseudo first and second order

*T* (°C)	Pseudo-first-order kinetics			Pseudo-second-order kinetics		
	*q*_e,cal_ (mg g^−1^)	*k*_1_ (min^−1^)	*R* ^2^	*q*_e,cal_ (mg g^−1^)	*k*_2_	*R* ^2^
25	39.24	0.1647	0.702	42.20	0.0057	0.845
35	45.41	0.1823	0.752	48.69	0.0056	0.885
45	54.16	0.0983	0.720	58.18	0.0026	0.854

### 3.4 Adsorption isotherms

**Fig. 10:**
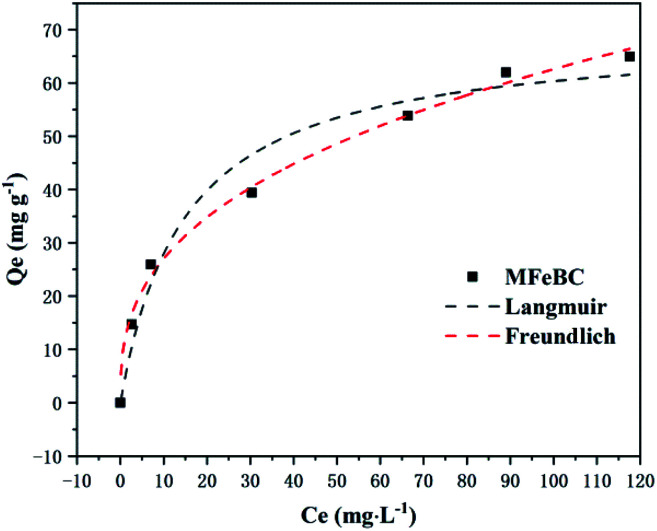
Adsorption isotherms for TC by MFeBC.

As shown in Fig. 10, the adsorption capacity increased with the increase of the initial TC concentration. To provide a better description of the adsorption mechanism, two typical adsorption models, Langmuir (eqn (5)) and Freundlich (eqn (6)) isotherm models were applied to simulate the adsorption isotherms of TC onto MFeBC, respectively. Related parameters are listed in Table 2.5
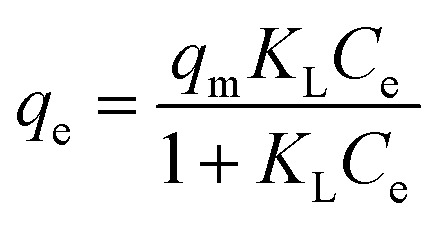

6 *q*_e_ = *K*_F_*C*_e_^1/*n*^

*C*_e_ (mg L^−1^) was the equilibrium concentration, *q*_e_ (mg g^−1^) was the TC adsorption capacity, *K*_L_ (L mg^−1^) was the Langmuir constant, *q*_m_ was the maximum adsorption capacity of the isotherm model, *K*_F_ (L g^−1^) was the Freundlich constant and 1/*n* was the empirical constant associated with adsorption intensity.^62^

**Table 2:** Adsorption isotherm model parameters for TC adsorption by MFeBC

Langmuir		Freundlich	
*q*_m_ (mg g^−1^)	69.26	*K*_F_	11.66
*K*_L_ (L mg^−1^)	0.068	1/*n*	0.3649
*R* ^2^	0.9473	*R* ^2^	0.9926

Adsorption isotherm is important for describing how the adsorbate molecules are distributed between the liquid and solid phases under an equilibrium state.^63^ The Langmuir isotherm assumed the adsorption occurs in the monolayer way with no interaction between the adsorbed molecular.^64^ Freundlich isotherm assumed the multilayer and nonideal adsorption on the heterogeneous surface with a non-uniform distribution of adsorption activation energy.^65^ Although the two typical isotherm models both fit the data well, Freundlich model exhibited a little better than Langmuir model. This may be caused by the introduction of Fe_3_O_4_ to the surface of biochar. In addition, the 1/*n* value (0.3649) defined as heterogeneity factor was less than 1 in Freundlich model. It was suggested that the interaction between adsorption sites and TC was *via* weak free energies, which was favorable to adsorption. This was consistent with other research.^66^ Other modified adsorbents were presented in Table 3. In our study, an efficient adsorbent material was prepared by pyrolysis at 300 °C for 1 h, greatly reducing the energy consumption and the cost of sewage treatment for the practical application of the possibility.

**Table 3:** Assessment on MFeBC's performance with other modified adsorbents for TC removal

Biochar feedstock	Preparation method	*T* (°C)	*Q*_m_ (mg g^−1^)	Ref.
Biogas residue	Citric acid modified	800 °C-2 h	20.77 mg g^−1^	68
Sawdust	Iron and zinc doped	600 °C-2 h	37.28 mg g^−1^	69
Rice husk	KOH modified	450–500 °C	58.8 mg g^−1^	70
Rice straw	Fe–N modified	700 °C	156 mg g^−1^	5
Commercial sawdust	Fe/Mn oxides loaded	500 °C-1 h	14.24	71
Crayfish shell	Ball milling	400 °C-2 h	39.1 mg g^−1^	72
Municipal sludge	Chitosan-Fe/S modified	500 °C-2h	51.78 mg g^−1^	73
Bagasse	HTC (230 °C-24 h) and heat treatment (400 °C-1 h)	230 °C-24 h	48.35 mg g^−1^	19
		400 °C-1 h		
Bagasse	Thiourea-functionalized magnetic biochar	300 °C-1 h	69.26 mg g^−1^	This work

Besides, the separation factor (*R*_L_) was always used to determine whether the adsorption was favorable or not. The *R*_L_ was expressed as follows (eqn (7)):7
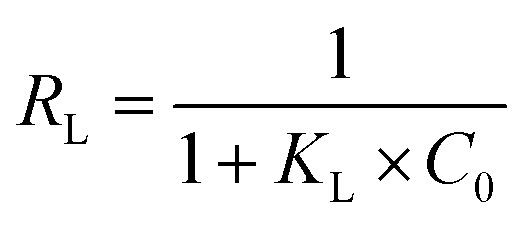
where *C*_0_ was the highest initial TC concentration (mg L^−1^) in this research. The value of *R*_L_ classified the nature of adsorption to be irreversible (*R*_L_ = 0), favorable (0 < *R*_L_ < 1), linear (*R*_L_ = 1) or unfavorable (*R*_L_ > 1).^67^ In this study, the *R*_L_ value was 0.0893, indicating that the adsorption was favorable.

## 4. Conclusions

An effective thiourea-functionalized magnetic biochar (MFeBC) was preparation *via* magnetization and functional modification for TC removal. N–S were successfully grafted on the surface of biochar. The modified biochar displayed that the maximum TC adsorption capacity of 69.26 mg g^−1^. The adsorbents could be fast separated. It was supposed that thiourea-introduced groups (sulfydryl and amidogen) and Π–Π interaction played a dominant role in TC adsorption process. The experimental results exhibited the adsorption data can be well fitted by Freundlich model and pseudo-second-order model. Our study provided a promising adsorbent with high adsorption capacity and good separation performance for TC wastewater treatment.

## Conflicts of interest

The authors declare that they have no known competing financial interests or personal relationships that could have appeared to influence the work reported in this paper.

## Supplementary Material
